# Volatile Organic Compound Fragmentation in the Afterglow of Pulsed Glow Discharge in Ambient Air

**DOI:** 10.3390/molecules27206864

**Published:** 2022-10-13

**Authors:** Denis Kravtsov, Anna Gubal, Victoria Chuchina, Natalya Ivanenko, Nikolay Solovyev, Alexander Stroganov, Han Jin, Alexander Ganeev

**Affiliations:** 1 Institute of Chemistry, St. Petersburg State University, Universitetskaya nab. 7/9, 199034 St. Petersburg, Russia; 2 Lumex Ltd., ul. Obruchevykh 1b, 195220 St. Petersburg, Russia; 3 Institute for Analytical Instrumentation of the Russian Academy of Sciences, ul. Ivana Chernykh, 31–33, lit. A, 198095 St. Petersburg, Russia; 4 School of Science, ATU Sligo, Atlantic Technological University, Ash Lane, F91 YW50 Sligo, Ireland; 5 Department of Instrument Science and Engineering, School of Electronic Information and Electrical Engineering, Shanghai Jiao Tong University, 800 Dongchuan Road, Shanghai 200240, China; 6 National Engineering Research Center for Nanotechnology, Shanghai 200241, China

**Keywords:** volatile organic compound, fragmentation, glow discharge, mass spectrometry

## Abstract

Glow discharge (GD) source gained an increased level of attention in relation to the analysis of volatile organic compounds (VOCs) since past work showed that this soft ionization method allowed direct analysis of VOCs with minimal fragmentation, however, the issue of fragmentation was not previously studied in detail. The aim of the present work was to investigate the effect of discharge conditions on VOC fragmentation in the system consisting of the cell with pulsed glow discharge and a time-of-flight mass spectrometer. Ionization of VOCs of different classes (hydrocarbons, alcohols, esters, and carboxylic acids) was investigated. A copper cathode with flat geometry was used. VOCs were ionized in the afterglow of short pulse glow discharge in the air. The use of discharge afterglow significantly reduces or eliminates the effects of ionization mechanisms other than Penning process, in particular, electron ionization. This significantly reduced VOC fragmentation and provided rather low limits of detection. Specific cluster formation was observed for alcohols and esters, which may facilitate their identification.

## 1. Introduction

Electron ionization (EI) is one of the most widely used ionization sources for mass spectrometric detection of various gaseous components, including volatile organic compounds (VOCs). An electron beam with set energy of e.g., 70 eV ionizes the molecules and the ions are then detected in a mass spectrometer [1]. EI is effective for the determination of elements and simple organic and inorganic molecules (N_2_, O_2_, CO, CO_2_, NO, NO_2_, etc.) in a gaseous phase. For VOCs, this ionization source causes significant fragmentation of the molecules, which considerably complicates data interpretation for the analysis of VOCs mixtures. Thus, alternative ionization sources are explored and developed. These include so-called chemical ionization (CI), including asymmetric charge transfer, protonation/deprotonation reactions, Penning ionization, and other processes [2,3,4]. Importantly, CI sources are usually selective and effectively ionize only a limited number of compounds. Fragmentation is also possible in CI, but it is considerably less common compared to EI.

Proton transfer mass spectrometry (PTR-MS) is capable of rapid and highly sensitive determination of VOCs without the use of complicated sample preparation protocols [5,6]. PTR-MS was first introduced in the late 1990s by Lindinger and co-workers at the University of Innsbruck, Austria [5,7]. In this source, the main ionization takes place through the protonation of VOC molecules by hydronium ion H_3_O^+^. The proton transfer takes place in a drift tube reactor, enabling the effective elimination of cluster ions. This simplifies both the proton transfer reaction and the mass spectrum data analysis. The exothermicity of the proton transfer reactions for the VOCs is relatively low. Thus, the degree of fragmentation is significantly lower than that for EI, while the masses of fragment ions are characteristic of the compounds of interest [8,9]. Nevertheless, a primary limitation of the technique is its inability to ionize alkanes and other compounds with low protonation potential. The use of fragment ions is mandatory for reliable identification [2]. Accordingly, the use of selective-reagent-ionization mass spectrometry (SRI-MS) [10] was proposed. This technique provides the possibility to switch between different reagent ions. For specific applications, different reagent ions (NO^+^, O_2_^+^, Kr^+^, and Xe^+^) were tested [10,11]. Limits of detection tend to ppq_v_. For more information about SRI-MS, please refer to [11,12]. The drawbacks of the technique are the artifacts, related to instrumental reason, memory effects and trace impurities present in noble gases. These issues were discussed in the study and possible solutions were offered [13]. Although the potentials of PTR-MS are quite considerable, its wide implementation is limited by the high costs of the switchable ionization mode instruments compared to older generation PTR-MS or the instruments with a GD source.

Selective ion flow tube mass spectrometry (SIFT-MS) may be considered a sister technique to PTR-MS. SIFT-MS quickly develops, exhibiting higher selectivity compared to PTR-MS, and is less affected by moisture in the sample [14]. The first difference is that reagent ions collide with reactive compounds to form product ions, which are well-known, because reagent ions are at thermal energy. Secondly, product ions and non-reacted reagent ions are carried down the flow tube by the carrier gas flow without an electrical field contrary to PTR-MS [2,3]. Usually, SIFT-MS maintains moist air plasma via a microwave discharge so that VOC molecules react with H_3_O^+^, NO^+^, and O_2_^+^ [3]. The most advanced SIFT-MS spectrometers may employ eight chemical ionizing agents (reagent ions): H_3_O^+^, NO^+^, O_2_^+^, O^−^, O_2_^−^, OH^−^, NO_2_^−^, and NO_3_^−^ [15,16]. These reagent ions react with VOCs but cannot react with primary air components (N_2_, O_2_, and Ar) and have a limited capacity to react with water vapor (which has lower reaction rates compared to VOCs). Therefore, the analysis may be undertaken with minimized sample preparation, and without preliminary concentrating, derivatization, and sample drying. Since main reagent ions react with the analytes in a different way, forming different association and fragmentation products, more structural information can be acquired. However, the efficiency of the reactant ion generation in the SIFT source is lower than that for the PTR-MS, which increases the limits of detection (LODs). Additionally, SIFT-MS is prone to considerable signal fluctuations, which necessitate regular instrument calibration, every few days. For instance, the study of Lehnert et al. [14] reported two-fold intensity and three-fold sensitivity drifts within a week.

Recently, glow discharge (GD) sources, formerly considered mainly as a source suitable for inorganic and isotopic analysis [17], have gained an increased level of attention in relation to the analysis of VOCs and volatile inorganic molecules mixtures [18]. Nunome et al. [4,19] reported the determination of VOCs in the air using a Soft Penning ionization (SPI) GD source. The pressure of 800–1200 Pa was demonstrated to result in mainly a Penning process ionization. However, a high level of fragmentation was observed for the analysis of smoke. This indicated incomplete separation between the Penning process and the discharge ionization *per se*. Under increased pressure of 1500–2000 Pa, the ionization was mainly switched to the NO^+^ association. The formation of [M + NO]^+^ associates shifts the analyte to the higher mass range, reducing potential interferences. However, the hydration of the associates [(M + NO)(H_2_O)_n_]^+^ complicates the identification.

For the determination of *n*-alkanes (C5-C14) and branched octanes, all compounds showed the formation of [M+O-3H]^+^ ions, except *n*-pentane and 2,2,4-trimethylpentane [20]. The authors achieved higher sensitivity compared to their previous study [4]. This improved the LODs to 0.124–1.68 ppm_v_ (determined by background ions), which is still not sufficient for practical applications. Besides, the interference of fragment ions and [M+O-3H]^+^ components may take place.

Fandino et al. [21] used a Grimm cell for the ionization of VOCs with the detection in a time-of-flight mass spectrometer (TOFMS). Radiofrequency (13.6 MHz) packets with a duration of 40–50 µs and packet repetition frequency of 400 Hz were used for the ionization. VOCs were detected in artificial air (a mixture of pure oxygen and nitrogen). Argon and the artificial air sample were separately introduced into the Grimm cell. Argon flow was pumped in next to the cathode surface; the artificial air containing VOCs was introduced into the hollow anode. This design partially separated the discharge zone and the zone of VOC ionization, reducing the fragmentation. Bouza et al. [22] used two variants of GD: as an ion source for mass spectrometry detection in gas chromatography and for direct input of VOCs (benzene, toluene, ethylbenzene, and xylene) in a flow of artificial air. VOCs were ionized in the discharge afterglow (99 µs after a pulse) due to the Penning process—the ionization of VOCs via collisions of VOC molecules with metastable atoms and molecules formed in GD plasma. The LOD values of VOCs in artificial air were ca. 1 ppb. Unfortunately, the detected fragmentation patterns of VOCs differed considerably from those available in the EI database.

It should be noted that a majority of previously published studies concern the determination of VOCs in artificial aerial mixtures. Certainly, artificial air is a simpler sample matrix compared to real ambient air. First of all, ambient air contains unstable quantities of moisture, which considerably affects all ionization mechanisms in the GD. The increase in sample humidity reduces the GD plasma electron temperature and, correspondingly, the concentration of metastable atoms and molecules, depressing the Penning ionization. Additionally, elevated humidity results in an increased probability of a protonation reaction of VOCs (M + H_3_O^+^ → MH^+^ + H_2_O), altering relative intensities in the mass spectra.

In 2020, a halo-flowing atmospheric-pressure afterglow ion source (h-FAPA) was used for the determination of VOCs [23]. This plasma-based ionization source was combined with an atmospheric pressure ionization time-of-flight mass spectrometer (API-TOFMS). Benzene was used as a model compound for direct input quantification of VOCs. The system showed high temporal stability (>40 min) in the formation of reactant ions, partially related to the thermal stability of modified h-FAPA. Oxygen and other oxidized species (e.g., pyrylium) were also observed in the mass spectra due to a special interface between the source and API-TOFMS. Since this is a brand new source type, tested only at a rather stable compound, it is currently hard to predict and evaluate the actual fragmentation of different groups of VOCs [23].

In our previous study [24], we used pulsed glow discharge in a copper hollow cathode coupled to a TOFMS for the quantification of VOCs as CuM^+^ associates. Under the discharge cell conditions used [24], a high concentration of copper atoms was maintained in the GD, which facilitated the formation of CuM species with their consequent ionization by two mechanisms. The first mechanism was the Penning process; the second one was related to the high-energy electron packet, forming on the front of the discharge pulse [25]. The use of CuM^+^ associates for the detection of VOCs shifts the analyte towards higher masses, where the intensities of background components are considerably lower. Besides, the di-isotopic pattern of copper serves as an additional identification feature of CuM^+^ species. The use of CuM^+^ was shown to considerably reduce the fragmentation of the compounds. The fragmentation of CuM species is mainly attributed to the formation of neutral Cu and M, so no new components are formed in the mass spectrum. However, the LODs of 0.2–0.8 ppm_v_ [24] limited the practical application of the approach. In a follow-up study, we managed to further reduce the LODs down to 0.5–5.0 ppb_v_ [18]. Air pulsed glow discharge coupled to the TOFMS was used, while molecular ions and their fragments were monitored as analytes in this case. A copper hollow cathode was used since, in the air GD, copper nearly did not form a non-conducting surface layer [18]. The achieved LODs benefited the real analytical application of the approach, in particular, for the determination of VOCs in human exhalation for diagnostic purposes [26]. Nevertheless, the challenge of the formation of a rather high level of copper-containing cluster ions complicates the identification and deteriorates the sensitivity for some compounds.

The aim of the present work was to investigate the effect of discharge conditions on VOC fragmentation. Penning ionization of VOCs in the afterglow of a short pulse glow discharge in the air–argon mixture was used. The ions were detected in the TOFMS. The use of discharge afterglow significantly reduces or eliminates the effects of ionization mechanisms other than the Penning process, in particular, EI. This significantly reduces VOC fragmentation. Note that Penning ionization of VOCs by metastable nitrogen molecules (N_2_*) provides a combination of high sensitivity and low fragmentation for a wide range of VOCs [18,24].

## 2. Results and Discussion

A mixture of three or four VOCs (chlorobenzene, toluene, *p-*xylene, and 1,2,4-trimethylbenzene) was used to optimize the discharge parameters. The optimization was undertaken for the same set of parameters as described previously [18,24]: discharge pulse duration, pressure, repelling pulse delay, and pulse period. The use of VOC mixtures allowed for accounting for different properties of the compounds such as binding energy, ionization energy, and saturated vapor pressure, affecting the relative intensities of the compounds. It is noteworthy that the effective ionization of VOCs was observed in a relatively narrow range of discharge parameters [18], see Section 2.4.

### 2.1. The Effect of Cathode Geometry

**Figure 1:**
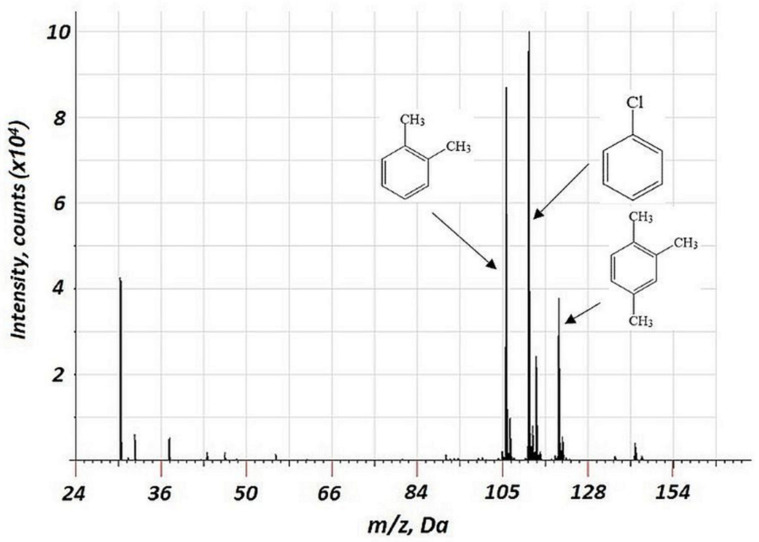
Background corrected mass spectrum range of 3 VOCs (chlorobenzene, *p-*xylene, and 1,2,4-trimethylbenzene) obtained using a flat copper cathode. Acquisition parameters were as follows: repelling pulse delay 200 µs, pulse duration 1.2 µs, pulse period 900 µs, discharge pressure 213 Pa; the concentration of all VOCs of 15 ppm.

The hollow cathode is known to trap the electron in the discharge, maintaining its energy and current density; electron energy in the hollow cathode exceeds that for the discharge with a flat cathode [27]. For elemental analysis of solid samples, both discharge cells with a combined hollow cathode and with a flat cathode (the so-called Grimm discharge cell and similar types of discharge cells) are used [28]. In our previous study, we used a copper hollow cathode and described the effective mechanism of VOC association with the materials of the cathode [18,24]. In this research, the flat geometry of a copper cathode was used There were no signs of cathode material-derived components in the mass spectra (Figure 1). This is probably related to the relatively high binding energy of the material. This is contrary to the previous data for the hollow copper cathode, the use of which results in a considerable formation of copper-containing cluster ions [24]. The absence of Cu-derived clusters in the mass spectrum may be related to the flat cathode geometry in the current study. The hollow cathode effect in the previous study may have resulted in the enhanced energy of ions, bombarding the cathode’s surface. For the flat geometry, the ion energy is lower.

### 2.2. The Comparison of the Performance for the Argon–Air Mixture and Air

**Figure 2:**
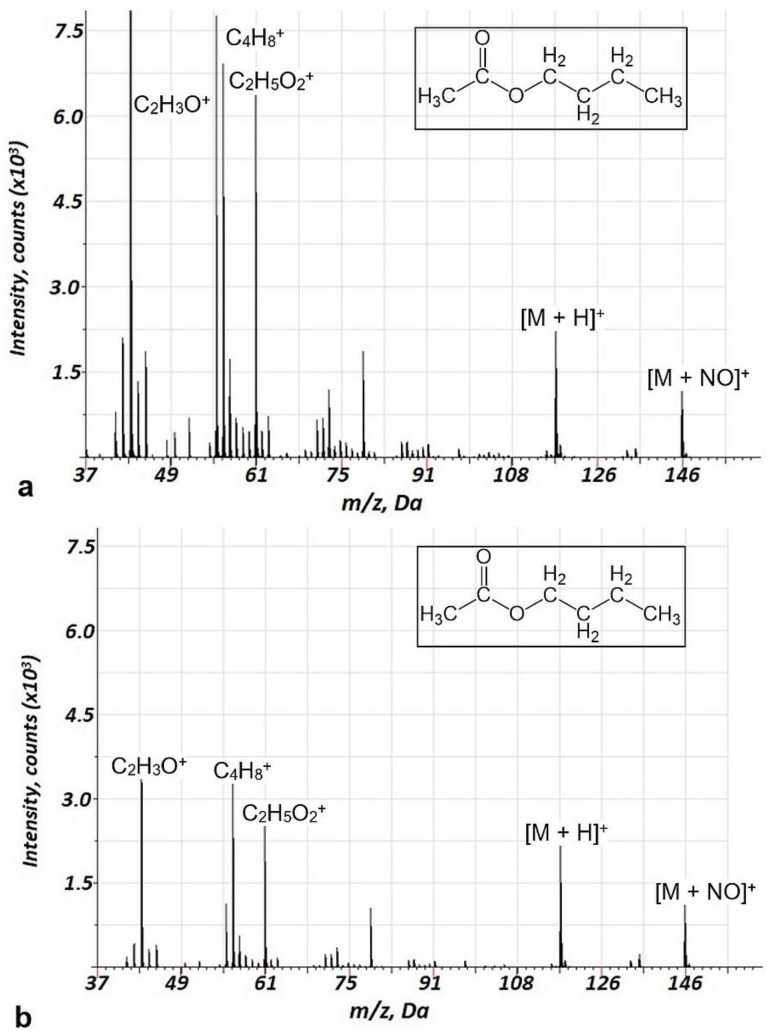
Background corrected mass spectrum ranges for butyl acetate in argon-air(**a**) and air (**b**) glow discharge under Penning ionization. Acquisition parameters were as follows: repelling pulse delay 200 µs, pulse duration 1.0 µs, pulse period 900 µs, (**a**) air pressure—110 Pa, Ar pressure—60 Pa, (**b**)—Air pressure—173 Pa; butyl acetate concentration of 20 ppm.

For the Penning ionization in GD, argon–air mixtures are usually used [3,8]. In our previous study [18], we showed the lower fragmentation in the argon–air mixture compared to pure Ar discharge gas. The advantages of this approach are high ionization efficiency and a low degree of fragmentation of VOCs. The reason for the low degree of fragmentation is related to the primary ionization mechanism of VOCs in the current study—the Penning process with metastable molecular nitrogen. In this study, we decided to use the direct flow of the air with VOCs instead of argon to reduce the costs of analysis and to increase sensitivity. The energy of argon’s metastable levels is 11.57 and 11.72 eV. For molecular nitrogen, this corresponds to 11.1 eV, which does not differ much from that of argon. Thus, Penning processes in argon–air mixtures and the air should be comparable. At the same time, binding energies for the varied groups of VOCs (Table S1) are in the range of 10.5–11.5 eV. Therefore, the fragmentation behavior of VOCs may differ considerably in the GD in the air and in the argon. This assumption was verified on several ester compounds. Mass spectra ranges for butyl acetate acquired in air and air–argon GD are shown in Figure 2. Analogous data for ethyl acetate and methyl acetate are presented in Supplementary Information (Figure S1 and Figure S2). Indeed, a reduced fragmentation in air discharge was observed compared to the argon–air plasma, especially for butyl acetate and ethyl acetate and to a lesser extent for methyl acetate.

### 2.3. Specific Fragmentation of Alcohols

Alcohol fragmentation in air GD was studied for ethanol, propanol-1, and butanol-1.

**Figure 3:**
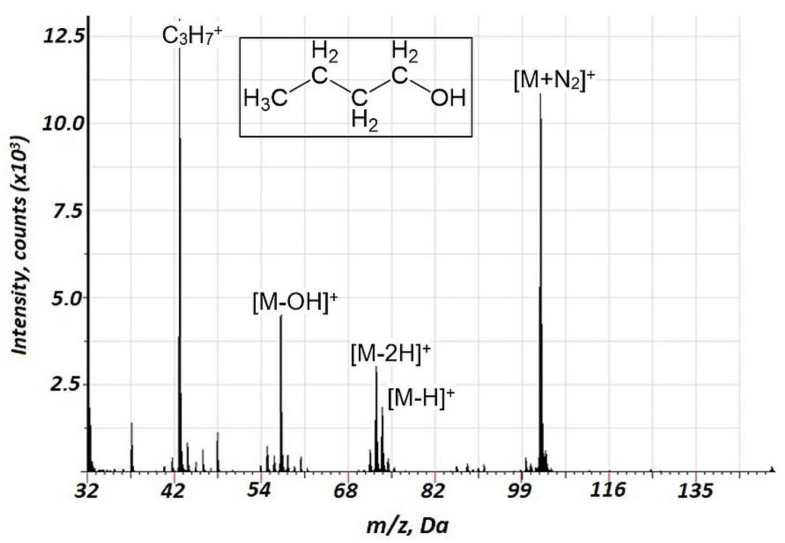
Background corrected mass spectrum range for butanol-1 obtained in air glow discharge. Acquisition parameters were as follows: discharge pulse duration 1.2 µs, discharge period 700 µs, discharge pressure 153 Pa, repelling pulse delay 100 µs; concentration of butanol-1 of 50 ppm.

Since the molecular ion of methanol ^12^C^1^H_3_^16^O^1^H^+^ interferes with ^16^O_2_^+^, methanol fragmentation was not studied. Fragmentation or reaction products for the interaction of VOCs with ^16^O_2_^+^ were not observed. The acquired mass spectra are presented in Figure 3 for butanol-1 (the data for propanol-1 and ethanol are presented in Figures S3 and S4, respectively, Supplementary Information). For the alcohols tested, MN_2_^+^ components were observed in the mass spectra together with mass spectral components present in the EI spectra. Interestingly, the relative intensities of MN_2_^+^ components for butanol-1 (Figure 3) and propanol-1 (Figure S3, Supplementary Information) significantly exceeded those for ethanol (Figure S4). Thus, there is an intriguing perspective of effective alcohol identification based on the MN_2_^+^ component formation that distinguishes it from the compounds of other classes. The source of the MN_2_^+^ species may be related to the following reaction, linked to the Penning mechanism:

M + N_2_* → MN_2_^+^ + *e*^−^ (1)

### 2.4. The Comparison of Penning Ionization in Glow Discharge and Electron Ionization

**Figure 4:**
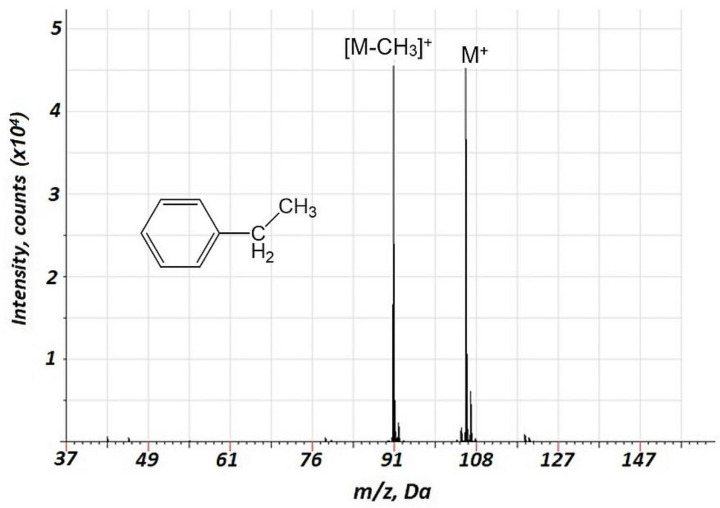
Background corrected mass spectrum range of ethylbenzene obtained using air glow discharge. Acquisition parameters were as follows: discharge pulse duration 1.2 µs, discharge period 900 µs, discharge pressure 153 Pa, repelling pulse delay 200 µs; concentration of ethylbenzene of 20 ppm.

**Figure 5:**
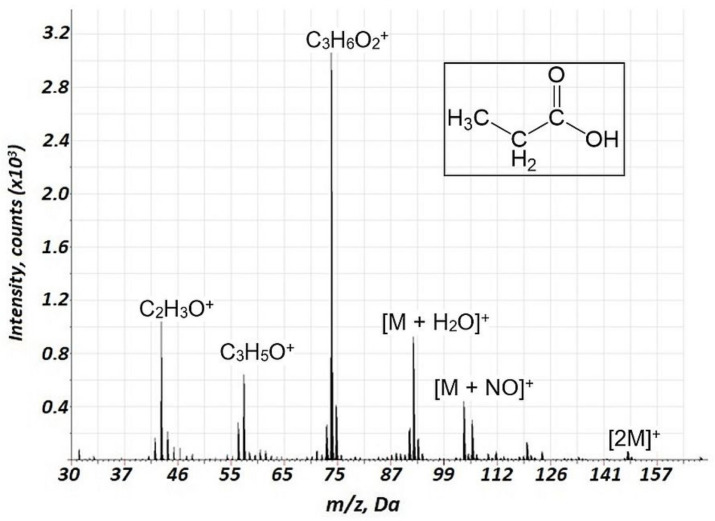
Background corrected mass spectrum range of propionic acid obtained using air glow discharge. Acquisition parameters were as follows: discharge pulse duration 1.1 µs, discharge period 900 µs, discharge pressure 133 Pa, repelling pulse delay 200 µs; concentration of propionic acid of 70 ppm.

Importantly, a considerably lower degree of fragmentation and a smaller number of fragments were observed in the case of air GD ionization in the current study compared to the EI of VOCs. This is illustrated in Table 1 (*n*-heptane, *n*-octane, ethylbenzene, propanol-1, and propionic acid), indicating relative intensities for the major mass spectrometric components for different classes of VOCs for the current approach and 70 eV EI (further detailed in Supplementary Table S2). For instance, for the fragmentation of alkanes, in particular, *n*-heptane and *n*-octane (Supplementary Figures S5 and S6, respectively) the intensities of molecular ions are rather high, especially for *n-*heptane. The primary fragment ions are [M-C_2_H_5_]^+^. Additionally, we investigated the mass spectra of ethylbenzene (Figure 4) and propionic acid (Figure 5), which are important biomarkers of lung cancer in humans [20]. For these two compounds, a considerably lower fragmentation compared to the EI (Table 1) was also obtained. For ethylbenzene, only two components were observed in the mass spectrum (Figure 4), one of which corresponded to the molecular ion. For propionic acid, together with reduced fragmentation, the formation of [M + H_2_O]^+^ and [M + NO]^+^ associates was observed.

Importantly, the range of discharge parameters (discharge pulse duration, the pressure of discharge gas, current, energy in the pulse, and total power) ensuring high sensitivity, low LODs, and relatively low degree of fragmentation, is rather narrow. In particular, discharge pulse duration in the range of 1.0–1.2 µs, air pressure of 130–160 Pa, and average power of 1–2 W ensured the best performance.

Under the tentatively optimized parameters, effective VOC ionization with low fragmentation and high intensity of molecular ions was combined with, in principle, a conflicting process of low sputtering and low ionization of the cathode material. The parameters optimized in the current study differed considerably from those reported in our previous study [24] and the results of other groups [4,20,21]. For our previous study, the average discharge power was 6–10 W, while in this study the power is only 1.5–3.0 W. Another considerable difference from the studies [24] and [21] is the possibility to introduce the analyzed air without its dilution with argon, significantly improving the sensitivity. The improved stability and a relatively low degree of fragmentation of VOCs ensured relatively low LODs of VOCs (Table 2).

Importantly, a considerably lower degree of fragmentation and a smaller number of fragments were observed in the case of air GD ionization in the current study compared to the EI of VOCs. This is illustrated in Table 2, indicating relative intensities for the major mass spectrometric components of different classes of VOCs for the current approach and 70 eV EI. Notably, for the alcohols, intensive ion clusters MN_2_^+^ were observed (Table 2, Figure 3, Figures S3 and S4) which may be used for the effective identification of these compounds. For the esters (acetates) and carboxylic acids, MNO^+^ clusters were observed (Table 2, Figure 2, Figure 5 and Figure S1), which may also be a perspective for ester identification.

The effect of humidity deviations was rather low, as was demonstrated also by Gubal et al. [24]. Under the determination of VOCs in ambient air, a 1.5-fold deviation in humidity resulted in only a 4–10% alteration in intensities of VOCs, which nearly corresponded to the random experimental error. For instance, for the analysis of exhaled air, the humidity fluctuates in the range of 0.9–1.0 of saturated water vapor; such humidity fluctuations may result in intensity drifts of ca. 2–5%. Thus, the effect of humidity can be neglected, which significantly facilitates the use of the designed approach for the analysis of human exhalation, which is rich in water vapor (approaches the saturated vapor pressure at r.t.). The absence of the pronounced interfering effect of water vapor is a significant advantage of the proposed air pulsed GD ionization source for the analysis of VOCs in the air.

### 2.5. Limits of Detection

The LODs, assessed using a 3σ criterion for the acquisition time of 10 min, are presented in Table 2. For the majority of compounds tested, the LOD values are in the range of 0.5–5 ppb. This facilitates a wide practical use of the proposed approach including testing human breath for VOCs presence.

Fandino et al. [21] achieved the LODs in the range of 100–200 ppb for arenes quantification in an argon discharge. It is noteworthy that currently achievable LODs are quite comparable to these obtained by PTR-MS (10–100 ppb) according to previous publications [29,30,31]. This demonstrates the prospects of further advancements in the GD ionization source in molecular mass spectrometry.

## 3. Materials and Methods

### 3.1. Instrumentation

A time-of-flight mass spectrometer Lumas-30 (Lumex, St. Petersburg, Russia) was used throughout this study. The ionization source was a µs-pulsed glow discharge. The resolution (*m*/Δ*m*) was ca. 800 in the mass range up to 350 Da. The device and its specifications were detailed previously [18,24]. The discharge cell consisted of the flat cathode and a mass spectrometer’s sampler serving as an anode. The cell was modified for the analysis of gaseous samples. The copper flat cathode (2 mm thick, made ofcopper foil, 99.999% purity) was tested in the current study: Ambient air or argon (99.9999%, Vossen, Moscow, Russia) was used as a discharge gas. Mass spectra were acquired at a delay time (*τ_i_*) of 1–250 µs after the discharge. The discharge frequency (*F*) was 1–3.2 kHz under the duration (*τ_d_*) of 1–4 µs. Final spectra were obtained through the summation of the spectra from separate packets. Background spectra were subtracted from all sample spectra; normalization was performed by macrocomponents of the spectra (NO, O_2_, and H_2_O). The intensity of isotope components was obtained by integrating the mass spectrum using an integration window of ± 0.15 Da from a peak center.

### 3.2. Samples and Sample Introduction

The different classes of VOCs **(***n-*heptane, *n-*octane, *n-*decane, toluene, ethylbenzene, *p-*xylene, 1,2,4-trimethylbenzene, chlorobenzene, isoamyl alcohol, isobutanol, butanol-1, propanol-1, ethanol, propionic acid, butyl acetate, ethyl acetate, methyl acetate) studied in the current work were at least HPLC grade (≥99.9%) and purchased from ChromLab (Lubertsy, Russia). A quartz capillary, installed into the argon flow line, was used for the sample introduction. The length of the capillary was 30–50 cm with an inner diameter of 140 µm. Samples were introduced from 3 L Tedlar^®^ bags (Restek, Centre County, PA, USA). The bag was filled in with laboratory air, after which 0.1–1.0 µL of VOC under study was injected into the bag using a microsyringe (Hamilton, Reno, NV, USA). To reduce the concentration of the VOC in the bag, dilution was used. The bag was pumped out with a known pumping rate down to 50–80% of the volume and was refilled again with laboratory air to full volume.

The bag was heated up with a technical fan for at least 2–3 min to achieve full evaporation. The capillary was introduced into the bag via a septum. The VOCs–air mixture or the air was pumped into the cell due to the pressure difference between the cell and the atmosphere. No valves were installed in the sample introduction system. The background spectrum of the ambient air was recorded prior to every measurement.

### 3.3. Statistics

Six replicates (*n* = 6) were undertaken in the case of all measurements, including the LOD assessment. All quantification results are presented as a mean ± confidence interval (*n* = 6, *p* = 0.95).

The LODs were evaluated using the following equation as reported previously [32,33].

LOD = 3⋅*C*⋅Δ*I*_*bg*_*/(I − I*_*bg*_*)* (2)

where *I*—integral intensity of the compound under study, *I_bg_*—background intensity in the range ±0.15 u from the peak center, Δ*I_bg_*—standard deviation of background intensity, *C*—concentration of the compound in the sample.

## 4. Conclusions

The Penning mechanism of VOC ionization in the afterglow of air pulsed glow discharge was demonstrated to be highly efficient, ensuring high sensitivity and low limits of detection. Effective Penning ionization was caused by metastable nitrogen molecules, which did not initiate significant fragmentation of the analytes. For the determination of alcohols, a rather intensive formation of MN_2_^+^ associate was observed, which may simplify the identification of these compounds using the proposed approach. We consider that the developed approach may acquire relevant applications in the fields of exhaled air diagnostics as well as in environmental monitoring and technological control.

## Figures and Tables

**Table 1:** Relative intensities of mass spectrometric components for different VOCs obtained in the current study by pulsed glow discharge time-of-flight mass spectrometry (PGD-TOFMS) and compared to 70 eV electron ionization (EI). For each compound, the highest intensity fragment is taken as 100%.

VOC	Molecular Weight, Da	*m*/*z*	PGD-TOFMS, Relative Intensities of Ions, %	EI, Relative Intensities of Ions, % [1]
Alkanes				
*n*-Heptane	100	100	67	15
		71	100	45
		70	35	18
		57	44	47
		56	35	27
		43	13	100
		42	8	25
*n*-Octane	114	114	21	6
		85	100	26
		71	62	20
		57	33	34
		43	27	100
		41	5	44
		29	3	27
Arenes				
Ethylbenzene	106	106	100	28
		91	100	100
		77	-	10
		65	-	11
		51	-	11
		39	-	7
Alcohols				
Propanol-1	60	60	2.5	7
		88	68 (MN_2_^+^)	-
		59	48	12
		58	100	0
		31	34	100
Carboxylic acids				
Propionic acid	74	74	100	100
		57	21	46
		45	5	90
		43	37	6
		29	4	85
		28	15	94
		27	-	63
		93	29 (M + H_3_O^+^)	-
		104	14 (MNO^+^)	-

**Table 2:** Limits of detection (LODs) of VOCs in the air. The measurement conditions were as follows: acquisition time 10 min, discharge pulse duration 1.1 µs, discharge period 900 µs, discharge pressure 133 Pa, repelling pulse delay 200 µs.

VOC	LODs, ppb
Toluene	2.0
Chlorobenzene	3.0
*p-*Xylene	0.5
1,2,4-Trimethylbenzene	5.0
*o*-Xylene	0.5
Butanol-1	6.0
Propanol-1	4.0
Ethanol	3.0
*n*-Heptane	2.0
Methyl acetate	1.0
Propyl acetate	1.5
Butyl acetate	4.0

## Data Availability

The data presented in this study are contained within this article and are supported by the data in the Supplementary Materials.

## Supplementary Materials

The following supporting information can be downloaded at: https://www.mdpi.com/article/10.3390/molecules27206864/s1, Figure S1. Background corrected mass spectrum range of ethyl acetate (70 ppm) obtained using a flat silicon cathode for the air–argon (a) and air (b) glow discharge. Acquisition parameters: repelling pulse delay 200 µs, pulse duration 1.0 µs, pulse period 900 µs, air pressure 160 Pa, argon pressure 33 Pa. Figure S2. Background corrected mass spectrum range of methyl acetate (70 ppm) obtained using a flat silicon cathode for the air–argon (a) and air (b) glow discharge. Acquisition parameters: repelling pulse delay 200 µs, pulse duration 1.0 µs, pulse period 900 µs, air pressure 160 Pa, argon pressure 33 Pa. Figure S3 Background corrected mass spectrum range of propanol-1 (50 ppm) obtained using flat copper cathode air glow discharge. Acquisition parameters: repelling pulse delay 100 µs, pulse duration 1.2 µs, pulse period 300 µs, air pressure 133 Pa. Figure S4. Background corrected mass spectrum range of ethanol (100 ppm) obtained with a flat copper cathode for air glow discharge (Penning ionization). Acquisition parameters: repelling pulse delay 100 µs, pulse duration 1.2 µs, pulse period 300 µs, air pressure 133 Pa. Figure S5. Background corrected mass spectrum of heptane (50 ppm) obtained using a flat copper cathode for air glow discharge. Acquisition parameters: repelling pulse delay 200 µs, pulse duration 1.2 µs, pulse period 900 µs, air pressure 133 Pa. Figure S6. Background corrected mass spectrum range of octane (70 ppm) obtained using a flat silicon cathode for air glow discharge (Penning ionization). Acquisition parameters: repelling pulse delay 200 µs, pulse duration 1.0 µs, pulse period 900 µs, air pressure 160 Pa, argon pressure 33 Pa. Table S1. Binding energies for some volatile organic compounds according to the database [1]. Table S2. Relative intensities of mass spectrometric components for different VOCs obtained in the current study by pulsed glow discharge time-of-flight mass spectrometry (PGD-TOFMS) and compared to 70 eV electron ionization (EI). For each compound, the highest intensity fragment is taken as 100%.

## Sample Availability

Samples are not available from the corresponding authors.

## References

[1] Linstrom P.J., Mallard W.G. (2021). NIST Standard Reference Database Number 69.

[2] Yuan B., Koss A.R., Warneke C., Coggon M., Sekimoto K., de Gouw J.A. (2017). Proton-Transfer-Reaction Mass Spectrometry: Applications in Atmospheric Sciences. Chem. Rev..

[3] Smith D., Španěl P. (2005). Selected ion flow tube mass spectrometry (SIFT-MS) for on-line trace gas analysis. Mass Spectrom. Rev..

[4] Nunome Y., Kodama K., Ueki Y., Yoshiie R., Naruse I., Wagatsuma K. (2018). Development of soft ionization using direct current pulse glow discharge plasma source in mass spectrometry for volatile organic compounds analysis. Spectrochim. Acta Part B At. Spectrosc..

[5] Lindinger W., Hansel A., Jordan A. (1998). On-line monitoring of volatile organic compounds at pptv levels by means of proton-transfer-reaction mass spectrometry (PTR-MS) medical applications, food control and environmental research. Int. J. Mass Spectrom. Ion Processes.

[6] Olivenza-León D., Mayhew C.A., González-Méndez R. (2021). Proton transfer reaction mass spectrometry investigations of phthalate esters via direct headspace sampling. Int. J. Mass Spectrom..

[7] Hansel A., Jordan A., Holzinger R., Prazeller P., Vogel W., Lindinger W. (1995). Proton transfer reaction mass spectrometry: On-line trace gas analysis at the ppb level. Int. J. Mass Spectrom. Ion Processes.

[8] De Gouw J.A., Goldan P.D., Warneke C., Kuster W.C., Roberts J.M., Marchewka M., Bertman S.B., Pszenny A.A.P., Keene W.C. (2003). Validation of proton transfer reaction-mass spectrometry (PTR-MS) measurements of gas-phase organic compounds in the atmosphere during the New England Air Quality Study (NEAQS) in 2002. J. Geophys. Res. Atmos..

[9] De Gouw J., Warneke C. (2007). Measurements of volatile organic compounds in the earth’s atmosphere using proton-transfer-reaction mass spectrometry. Mass Spectrom. Rev..

[10] Jordan A., Haidacher S., Hanel G., Hartungen E., Herbig J., Märk L., Schottkowsky R., Seehauser H., Sulzer P., Märk T.D. (2009). An online ultra-high sensitivity Proton-transfer-reaction mass-spectrometer combined with switchable reagent ion capability (PTR+SRI−MS). Int. J. Mass Spectrom..

[11] Sulzer P., Edtbauer A., Hartungen E., Jürschik S., Jordan A., Hanel G., Feil S., Jaksch S., Märk L., Märk T.D. (2012). From conventional proton-transfer-reaction mass spectrometry (PTR-MS) to universal trace gas analysis. Int. J. Mass Spectrom..

[12] Müller M., Piel F., Gutmann R., Sulzer P., Hartungen E., Wisthaler A. (2020). A novel method for producing NH4+ reagent ions in the hollow cathode glow discharge ion source of PTR-MS instruments. Int. J. Mass Spectrom..

[13] Salazar Gómez J.I., Klucken C., Sojka M., Masliuk L., Lunkenbein T., Schlögl R., Ruland H. (2019). Elucidation of artefacts in proton transfer reaction time-of-flight mass spectrometers. J. Mass Spectrom..

[14] Lehnert A.S., Behrendt T., Ruecker A., Pohnert G., Trumbore S.E. (2020). SIFT-MS optimization for atmospheric trace gas measurements at varying humidity. Atmos. Meas. Tech..

[15] Langford V.S., Padayachee D., McEwan M.J., Barringer S.A. (2019). Comprehensive odorant analysis for on-line applications using selected ion flow tube mass spectrometry (SIFT-MS). Flavour Fragr. J..

[16] Hastie C., Thompson A., Perkins M., Langford V.S., Eddleston M., Homer N.Z.M. (2021). Selected Ion Flow Tube-Mass Spectrometry (SIFT-MS) as an Alternative to Gas Chromatography/Mass Spectrometry (GC/MS) for the Analysis of Cyclohexanone and Cyclohexanol in Plasma. ACS Omega.

[17] Gubal A., Chuchina V., Sorokina A., Solovyev N., Ganeev A. (2021). Mass spectrometry-based techniques for direct quantification of high ionization energy elements in solid materials—challenges and perspectives. Mass Spectrom. Rev..

[18] Gubal A., Chuchina V., Lyalkin Y., Ivanenko N., Solovyev N., Stroganov A., Ganeev A. (2021). New Possibilities for the Determination of Volatile Organic Compounds by Their Molecular Ions in Air Using µs-Pulsed GD TOFMS. At. Spectrosc..

[19] Nunome Y., Park H., Kodama K., Ueki Y., Yoshiie R., Lee S.C., Kitagawa K., Wagatsuma K., Naruse I. (2015). Use of Soft Plasma Ionization Source at Evacuated Air Atmospheres in Time-of-Flight Mass Spectrometry to Suppress Fragmentation of Volatile Organic Compounds. Spectrosc. Lett..

[20] Nunome Y., Kodama K., Ueki Y., Yoshiie R., Naruse I., Wagatsuma K. (2019). Direct analysis of saturated hydrocarbons using glow discharge plasma ionization source for mass spectrometry. Talanta.

[21] Fandino J., Bouza M., Pisonero J., Blanco D., Sanz-Medel A., Bordel N. (2018). A novel gas sampling introduction interface for fast analysis of volatile organic compounds using radiofrequency pulsed glow discharge time of flight mass spectrometry. Anal. Chim. Acta.

[22] Bouza M., Fandino J., Bordel N., Pereiro R., Sanz-Medel A. (2017). Volatile organic compound analysis by pulsed glow discharge time of flight mass spectrometry as a structural elucidation tool. J. Mass Spectrom..

[23] Fandino J., Orejas J., Chauvet L., Blanco D., Guillot P., Pisonero J., Bordel N. (2020). Evaluation of a modified halo flowing atmospheric pressure afterglow ion source for the analysis of directly injected volatile organic compounds. J. Anal. At. Spectrom..

[24] Gubal A., Chuchina V., Ivanenko N., Qian R., Solovyev N., Ganeev A. (2020). Microsecond pulsed glow discharge in copper hollow cathode reveals a new approach to ionization and determination of volatile organic compounds. Spectrochim. Acta Part B At. Spectrosc..

[25] Bodnar V., Ganeev A., Gubal A., Solovyev N., Glumov O., Yakobson V., Murin I. Pulsed glow discharge enables direct mass spectrometric measurement of fluorine in crystal materials—Fluorine quantification and depth profiling in fluorine doped potassium titanyl phosphate. Spectrochim. Acta B.

[26] Ganeev A.A., Gubal A.R., Lukyanov G.N., Arseniev A.I., Barchuk A.A., Jahatspanian I.E., Gorbunov I.S., Rassadina A.A., Nemets V.M., Nefedov A.O. (2018). Analysis of exhaled air for early-stage diagnosis of lung cancer: Opportunities and challenges. Russ. Chem. Rev..

[27] Moskalev B. (1969). Discharge with a Hollow Cathode.

[28] Gubal A., Ganeev A., Hoffmann V., Voronov M., Brackmann V., Oswald S. (2017). Combined hollow cathode vs. Grimm cell: Semiconductive and nonconductive samples. J. Anal. At. Spectrom..

[29] Barber S., Blake R.S., White I.R., Monks P.S., Reich F., Mullock S., Ellis A.M. (2012). Increased Sensitivity in Proton Transfer Reaction Mass Spectrometry by Incorporation of a Radio Frequency Ion Funnel. Anal. Chem..

[30] Yadav R., Beig G., Anand V., Kalbande R., Maji S. (2022). Tracer-based characterization of source variations of ambient isoprene mixing ratios in a hillocky megacity, India, influenced by the local meteorology. Environ. Res..

[31] Müller M., Mikoviny T., Feil S., Haidacher S., Hanel G., Hartungen E., Jordan A., Märk L., Mutschlechner P., Schottkowsky R. (2014). A compact PTR-ToF-MS instrument for airborne measurements of volatile organic compounds at high spatiotemporal resolution. Atmos. Meas. Tech..

[32] Murray Kermit K., Boyd Robert K., Eberlin Marcos N., Langley G.J., Li L., Naito Y. (2013). Definitions of terms relating to mass spectrometry (IUPAC Recommendations 2013). pac.

[33] Marcus R.K., Broekaert J.A.C. (2003). Glow Discharge Plasmas in Analytical Spectroscopy.

