# Supplementary material for: Volatile Organic Compound Fragmentation in the Afterglow of Pulsed Glow Discharge in Ambient Air
Source: Molecules. 2022 Oct 13;27(20):6864. doi: 10.3390/molecules27206864 (PMC9611247; doi:10.3390/molecules27206864)
Supplement: Supplementary file 1 [file molecules-27-06864-s001.zip › molecules-1962527 Supplementary.pdf]

SUPPLEMENTARY INFORMATION

Volatile organic compound fragmentation in the afterglow of pulsed glow discharge in ambient air

Denis Kravtsov[1,2,3], Anna Gubal[1,2], Victoria Chuchina[1,2,*], Natalya Ivanenko[2], Nikolay Solovyev[4], Alexander Stroganov[2], Han Jin[5,6], Alexander Ganeev[1,2]

[1] Institute of Chemistry, St. Petersburg State University, Universitetskaya nab. 7/9, 199034 St. Petersburg, Russia

[2] Lumex Ltd., ul. Obruchevykh 1b, 195220 St. Petersburg, Russia

[3] Institute for Analytical Instrumentation of the Russian Academy of Sciences, ul. Ivana Chernykh, 31–33, lit. A, 198095 St. Petersburg, Russia

[4] School of Science, ATU Sligo, Atlantic Technological University, Ash Lane, F91 YW50 Sligo, Ireland

[5] Department of Instrument Science and Engineering, School of Electronic Information and Electrical Engineering, Shanghai Jiao Tong University, 800 Dongchuan Road, Shanghai 200240, China

[6] National Engineering Research Center for Nanotechnology, Shanghai 200241, China

* Correspondence: v.chuchina@spbu.ru

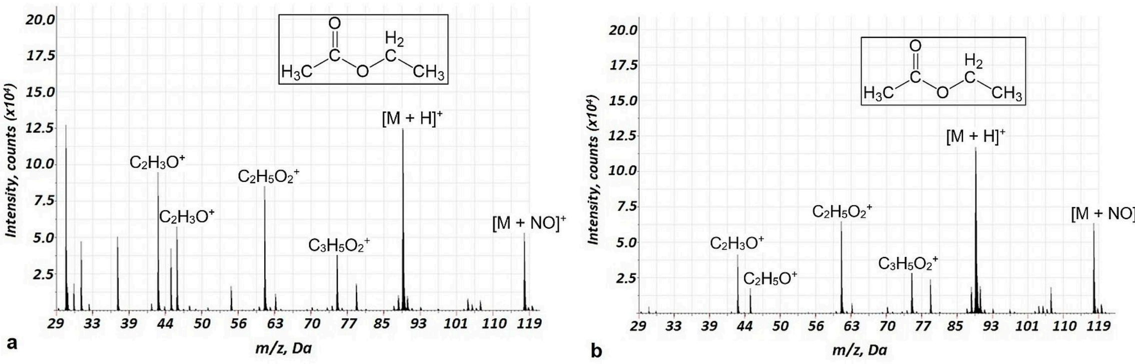

Figure S1. Background corrected mass spectrum range of ethyl acetate (70 ppm) obtained using a flat silicon cathode for the air-argon (a) and air (b) glow discharge. Acquisition parameters: repelling pulse delay 200 µs, pulse duration 1.0 µs, pulse period 900 µs, air pressure 160 Pa, argon pressure 33 Pa.

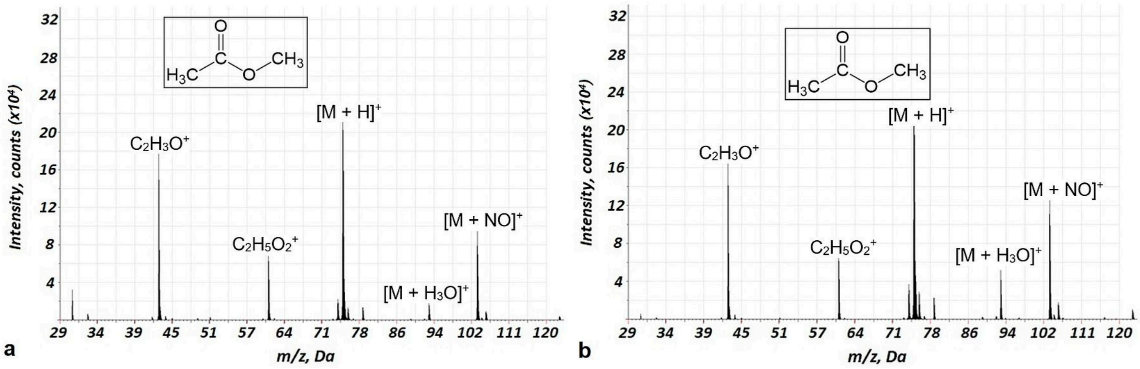

Figure S2. Background corrected mass spectrum range of methyl acetate (70 ppm) obtained using a flat silicon cathode for the air-argon (a) and air (b) glow discharge. Acquisition parameters: repelling pulse delay 200 µs, pulse duration 1.0 µs, pulse period 900 µs, air pressure 160 Pa, argon pressure 33 Pa.

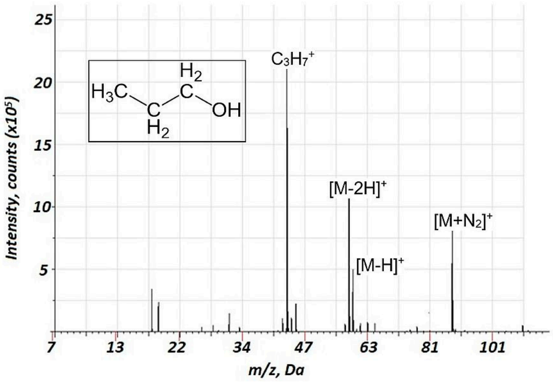

Figure S3 Background corrected mass spectrum range of propanol-1 (50 ppm) obtained using flat copper cathode air glow discharge. Acquisition parameters: repelling pulse delay 100 μs, pulse duration 1.2 μs, pulse period 300 μs, air pressure 133 Pa.

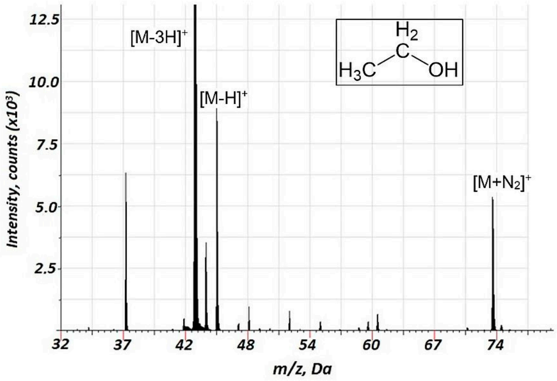

Figure S4. Background corrected mass spectrum range of ethanol (100 ppm) obtained with a flat copper cathode for air glow discharge (Penning ionization). Acquisition parameters: repelling pulse delay 100 μs, pulse duration 1.2 μs, pulse period 300 μs, air pressure 133 Pa.

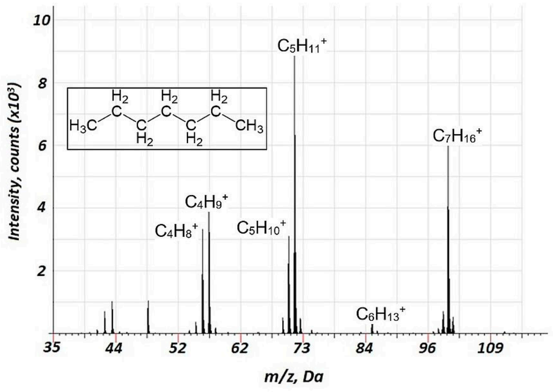

Figure S5. Background corrected mass spectrum of heptane (50 ppm) obtained using a flat copper cathode for air glow discharge. Acquisition parameters: repelling pulse delay 200 μs, pulse duration 1.2 μs, pulse period 900 μs, air pressure 133 Pa.

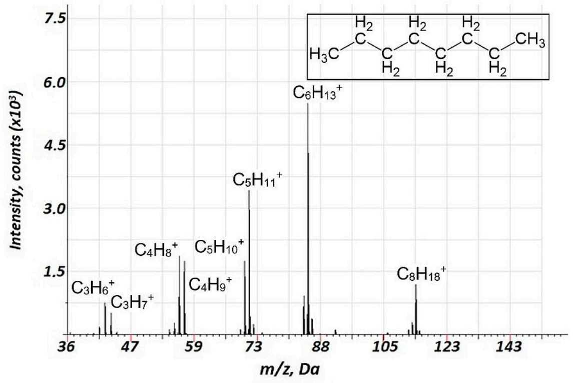

Figure S6. Background corrected mass spectrum range of octane (70 ppm) obtained using a flat silicon cathode for air glow discharge (Penning ionization). Acquisition parameters: repelling pulse delay 200 μs, pulse duration 1.0 μs, pulse period 900 μs, air pressure 160 Pa, argon pressure 33 Pa.

Table S1. Binding energies for some volatile organic compounds according to NIST database [1].

Compound	Ionization energy, eV	Fragment	Energy, eV	Residue
		**Alkanes**		
**n-Hexane**	10.2	$C_3H_6^+$	11	C_3H_8
		$C_3H_7^+$	11.4	C_3H_7
		$C_4H_8^+$	11	C_2H_6
		$C_4H_9^+$	11.1	C_2H_5
		$C_5H_{10}^+$	11	CH_4
		$C_5H_{11}^+$	11.1	CH_3
**Cyclohexane**	9.9	$C_3H_6^+$	11.2	C_3H_6
		$C_3H_7^+$	11.5-13.5	C_3H_5
		$C_4H_7^+$	11.2	C_2H_5
		$C_4H_8^+$	11.1-11.5	C_2H_4
		$C_5H_9^+$	9.9-11.2	CH_3
		$C_6H_{11}^+$	11.3-11.7	H
**n-Heptane**	9.9	$C_3H_6^+$	10.7 -10.9	C_4H_{10}
		$C_3H_7^+$	11.1-11.6	No data*
		$C_4H_7^+$	11.5	No data
		$C_4H_8^+$	10.6-11.0	C_3H_8
		$C_4H_9^+$	10.6-11.2	C_3H_7
		$C_5H_{10}^+$	10.3-11.0	C_2H_6
		$C_5H_{11}^+$	10.4-11.0	C_2H_5
		$C_6H_{12}^+$	11.2	CH_4
		$C_6H_{13}^+$	10.7 -10.9	CH_3
**n-Octane**	9.8	$C_3H_7^+$	11.9	No data
		$C_4H_8^+$	11.1-11.4	C_4H_{10}/n-C_4H_9No data
		$C_5H_{10}^+$	11.1	C_3H_8
		$C_5H_{11}^+$	11.0-11.2	C_3H_7

		$C_6H_{12}^+$	10.3-10.8	C_2H_6
		$C_6H_{13}^+$	10.9	C_2H_5
		$C_7H_{15}^+$	10.9	CH_3
**n-Nonane**	10.2	$C_4H_9^+$	11.2	No data
		$C_5H_{11}^+$	11.1	C_4H_9
		$C_6H_{13}^+$	10.6	No data
**n-Decane**	9.7		No data	
**n-Undecane**	9.6		No data	
**n-Dodecane**	10.4	$C_6H_{12}^+$	10.4	No data
		**Alcohols**		
**Methanol**	10.84		> 12	
**Ethanol**	10.47	CH_3O^+	11.2-11.4	CH_3
		$C_2H_5O^+$	10.6-10.8	H
**Propanol-1**	10.15	CH_3O^+	11.1-12.3	C_2H_5
		$C_2H_5O^+$	11.1-11.4	CH3
		$C_3H_6^+$	10.3-10.7	H2O
		$C_3H_7O^+$	10.2-10.7	H
**Propanol-2**	10.1	$C_2H_4O^+$	10.2-10.3	CH_4
		$C_2H_5O^+$	10.2-10.7	CH_3
		$C_3H_7O^+$	10.3-11.9	H
**Butanol-1**	10.04	CH_3O^+	11.4	n- C_3H_7
		$C_2H_2O^+$	11.2	No data
		$C_4H_8O^+$	10.2	H_2O
**Isobutanol**	10.12	CH_5O^+	10.4-10.5	C_3H_5
		$C_3H_6^+$	11.0 -11.3	CH_3OH
		$C_4H_8^+$	10.3	H_2O
**Butanol-2**	10.1	$C_2H_4O^+$	10.1	C_2H_6
		$C_2H_5O^+$	10.2-10.4	C_2H_5
		$C_3H_6O^+$	10.2	CH_4
		$C_3H_7O^+$	10.1-10.7	CH_3
		$C_3H_8O^+$	10.2	CH_4
**Pentanol-1**	10.38	$C_5H_{10}^+$	10	H_2O
**Pentanol-2**	9.78	$C_2H_5O^+$	10.2	1- C_3H_7
**Isoamyl aclohol**	No data		No data	
**Hexanol-1**	10.35	$C_2H_4O^+$	10.7	No data
		$C_4H_8O^+$	9.9	No data

**Benzyl alcohol**	9.23	$C_6H_7^+$	10.3-10.9	No data
		$C_7H_7O^+$	10.0-10.5	No data
		**Carbonyle compounds**		
**Formaldehyde**	10.9	CHO^+	11.9	H
**Ethanal**	10.2	$C_2H_2O^+$	10.7 -13.1	H_2
		$C_2H_3O^+$	10.5-11.0	H
**Propanal**	9.9	$C_2H_3O^+$	10.8	CH_3
		$C_3H_5O^+$	10.2	H
**Butanal**	9.8	$C_2H_3O^+$	10.2	C_2H_5
		$C_2H_4O^+$	10.5	C_2H_4
		$C_3H_5O^+$	10.2	No data
**Pentanal**	9.7	$C_2H_4O^+$	11.4	C_3H_6
		$C_3H_6^+$	11.9	C_2H_4O
		$C_3H_6O^+$	9.8-10.0	C_2H_4
		$C_5H_8^+$	9.8-10.0	H_2O
**Hexanal**	9.8	$C_2H_4O^+$	11.6	C_4H_8
		$C_3H_6O^+$	10.2	C_3H_6
		$C_3H_6O^+$	9.7	C_3H_6
		$C_4H_8^+$	10.7	C_2H_4O
		$C_4H_8O^+$	10	C_3H_6
		$C_6H_{10}O^+$	9.8	H_2O
**Heptanal**	9.65		No data	
**Acetone**	9.7	$C_2H_2O^+$	10.7	CH_4
		$C_2H_3O^+$	10.3-12.2	CH_3
		**Carboxylic acids**		
**Formic acid**	11.1		> 12	
**Acetic acid**	10.7	$C_2H_3O^+$	11.4-11.8	OH
**Propanoic acid**	10.2	$C_3H_4O^+$	10.9-11.6	H_2O
		$C_3H_5O^+$	11.0-12.2	OH
		$C_3H_5O_2^+$	11.1-11.7	H
**Butanoic acid**	10.2	$C_2H_4^+$	11.5	$C_2H_4O_2$
		$C_2H_4O_2^+$	10.4-10.6	C_2H_4
		$C_2H_5^+$	11.6	$C_2H_3O_2$
		$C_2H_40_2^+$	10.6	C_2H_4
		$C_3H_5O_2^+$	10.5	CH_3

		$C_3H_6^+$	11.4	CH_2O_2
		$C_3H_7^+$	11	COOH
		$C_4H_7O^+$	11.8	OH
		**Arenes**		
**Benzene**	9.2		> 12	
**Toluene**	8.8	$C_7H_7^+$	10.7-11.8	H
		$C_7H_7^+[C_6H_5CH_2^+]$	10.7 -11.2	H
		$C_7H_7^+$ [c- $C_7H_7^+$]	10.5-11.1	H
**Ethylbenzene**	8.8	$C_6H_6^+$	11	C_2H_4
		$C_7H_7^+$	9.9-10.9	CH_3
		$C_8H_9^+$	10.6-12.1	H
**o-Xylene**	8.6	$C_7H_7^+$	11.1-11.8	CH_3
		$C_8H_9^+$	11.3-12.1	H
**m-Xylene**	8.6	$C_7H_7^+$	11.3-11.8	CH_3
		$C_8H_9^+$	11.7-12.3	H
**p-Xylene**	8.4	$C_7H_7^+$	11.1-11.9	CH_3
		$C_8H_9^+$	11.4-12.1	H
**1,2,3-trimethylbenzene**	8.4		No data	
**1,2,4-trimethylbenzene**	8.3		No data	
**Chlorobenzene**	9.1	$C_6H_5^+$		Cl
**1,2,-Ddichlorobenzene**	9.1	> 12	11.8-13.2	
		**Esters**		
**Methyl acetate**	10.3	$C_2H_2O^+$	11.81 ± 0.15	No data
		$C_2H_3O^+$	10.9-11.4	CH_3O
		$C_2H_3O_2^+$	11.3-12.35	CH_3
**Ethyl acetate**	10.0	$C_2H_3O^+$	11.0-11.75	C_2H_5O
		$C_2H_3O_2^+$	11.74	C_2H_5
		$C_2H_4O_2^+$	11.2 ± 0.1	C_2H_4
		$C_2H_5^+$	11.3 -12.1	$C_2H_3O_2$
		$C_2H_5O^+$	10.7-10.8	C_2H_3O/CH_3CO
		$C_2H_5O_2^+$	10.6-11.0	C_2H_3
		$C_3H_5O_2^+$	10.6-11.0	CH_3

		$C_4H_6O^+$	10.3 -10.5	H_2O
**Propyl acetate**	9.9	$C_2H_5O_2^+$	9.9-10.5	CH_2=$CHCH_2$
		$C_3H_5O_2^+$	11.29 ± 0.04	C_2H_5
		$C_3H_7^+$	11.41 ± 0.04	No data
		$C_3H_7O^+$	11.64 ± 0.03	CH_3CO
**Butyl acetate**	10.0	$C_3H_5O_2^+$	11.70 ± 0.05	No data
		$C_3H_7^+$	11.56 ± 0.10	No data
		$C_4H_9^+$	11.31 ± 0.10	No data
**N,N-dimethylformamide**	9.1	$C_2H_6N^+$	11.6	No data
		$C_3H_6NO^+$	11.35	No data

Table S2. Relative intensities of mass spectrometric components for different VOCs obtained in the current study by pulsed glow discharge time-of-flight mass spectrometry (PGD-TOFMS) and compared to 70 eV electron ionization (EI). For each compound, the highest intensity fragment is taken as 100%.

VOC	Molecular weight, Da	*m/z*	PGD-TOFMS, relative intensities of ions, %	EI, relative intensities of ions, % [1]
			Alkanes	
n-Heptane	100	100	67	15
		71	100	45
		70	35	18
		57	44	47
		56	35	27
		43	13	100
		42	8	25
n-Octane	114	114	21	6
		85	100	26
		71	62	20
		57	33	34
		43	27	100
		41	5	44
		29	3	27
n-Decane	142	142	100	7
		141	71	-
		113	31	5
		99	44	8
		85	67	31
		71	56	44
		57	100	100
		55	71	32
			Arenes	
Toluene	92	92	100	78
		91	4	100
		78	1	-
Ethylbenzene	106	106	100	28
		91	100	100
		77	-	10
		65	-	11
		51	-	11

		39	-	7
p-Xylene	106	106	100	66
		91	8	100
		107	10	7
1,2,4-Trimethylbenzene	120	120	100	45
		121	10	4
		119	1	13
		105	2	100
Chlorobenzene	112, 114	112	100	100
		114	25	33
		113	4	7
		115	1	2
		77	-	45
		51	-	12
		50	-	10
Alcohols				
Ethanol	46	45	42	51
		43	100	12
		31	15	100
		29	-	30
		27	-	23
		74	30 (MN_2^+)	-
Propanol-1	60	60	2.5	7
		88	68 (MN_2^+)	-
		59	48	12
		58	100	0
		31	34	100
Isobutanol	74	102	3 (MN_2^+)	-
		88	55 (MN^+)	-
		74	2	14
		71	62	0.2
		58	20	0.8
		55	-	10
		43	100	100
		41	3	76
		33	-	32
		31	1	37
		29	-	15
Butanol-1	74	73	22	1.5
		72	35	-
		56	42	100
		43	100	69
		41	-	83
		31	12	98
		27	-	57
		102	84 (MN_2^+)	-
Isoamyl alcohol	88	87	18	0.75
		85	18	0.1
		105	12 (MOH^+)	-
		70	100	70
		58	21	1.5
		57	8	31

		55	11	100
		42	15	85
		39	-	45
		31	4	61
		29	2	60
		27	-	60
		Carboxylic acids		
Propionic acid	74	74	100	100
		57	21	46
		45	5	90
		43	37	6
		29	4	85
		28	15	94
		27	-	63
		93	29 ($M+H_3O^+$)	-
		104	14 (MNO^+)	-
		Esters		
Methyl acetate	74	104	60 (MNO^+)	-
		93	24 ($M+H_3O^+$)	-
		75	100	1
		74	17	25
		61	30	-
		59	-	11
		43	77	100
		29	-	7
Ethyl acetate	88	118	56 (MNO^+)	-
		107	15 ($M+H_3O^+$)	-
		89	100	0.6
		88	16	7
		79	20	-
		73	1	5
		70	4	12
		61	56	15
		45	16	15
		43	36	100
		29	1	12.5
Butyl acetate	116	146	37 (MNO^+)	-
		135	7 ($M+H_3O^+$)	-
		117	7	-
		115	2	0.1
		98	4	0.1
		87	4	1.7
		79	31	-
		73	13	18
		61	76	14
		56	100	37
		43	100	100

References:

[1] Linstrom P J and Mallard W G 2021 NIST Standard Reference Database Number 69. (National Institute of Standards and Technology, Gaithersburg MD p 20899
